# Canine parvovirus type 2 infection in vaccinated puppies: role of vaccination practices and viral antigenic variation

**DOI:** 10.1186/s12917-026-05403-0

**Published:** 2026-03-26

**Authors:** Mahmoud S. Safwat, Mohamed H. Khodeir, Rabie H. Etman

**Affiliations:** 1 Department of Internal Medicine and Infectious Diseases, Faculty of Veterinary Medicine, Cairo University, Giza, 12211 Egypt; 2 Department of Pet Animal Vaccine Research, Veterinary Serum and Vaccine Research Institute (VSVRI), Agriculture Research Center (ARC), Abassia 131, Cario, Egypt

**Keywords:** Canine parvovirus type 2, Egypt, Maternally derived antibodies, Modified live virus vaccine, MLV, Molecular characterization, Primary core vaccination series, VP2

## Abstract

**Background:**

Canine parvovirus type 2 (CPV-2) infection is reported in vaccinated puppies in Egypt, yet contributing factors remain poorly investigated. This study evaluated the role of vaccination practices and CPV-2 antigenic variation in disease occurrence in puppies whose primary vaccination series was recorded as finished by attending veterinarians.

**Methods:**

Puppies with clinical signs of parvoviral enteritis at three veterinary clinics in Giza, Egypt (June–October 2020) were enrolled if vaccination history was documented. All non-vaccinated puppies were included, whereas among vaccinated puppies, only those with a stamped finished primary vaccination series regardless of whether international guidelines had been followed were included. Rectal swabs were collected for PCR and VP2 gene sequencing. For vaccinated puppies, associations between PCR positivity and different aspects of vaccination practices, including age at finishing the vaccination series, number of doses, and vaccinal strain, were assessed. The finishing age was categorized as recommended (the final dose was given at ≥ 16 weeks of age, according to international guidelines) or early (< 16 weeks of age). CPV-2 variant distribution among vaccinated and non-vaccinated puppies was also evaluated.

**Results:**

Fifty-eight puppies met the inclusion criteria (41 vaccinated, 17 non-vaccinated). CPV-2 was PCR-positive in 28/41 vaccinated and 13/17 non-vaccinated puppies. Early finishing of the primary vaccination series was significantly associated with CPV-2 infection (*P* < 0.001), whereas vaccinal strain and number of doses were not. Disease developed within one month of vaccination, including six puppies within one week. Sequencing identified 35 new CPV-2a, 3 CPV-2b, and 3 CPV-2c variants, with no significant difference in variant distribution between vaccinated and non-vaccinated puppies (*P* = 0.16).

**Conclusions:**

Finishing the primary vaccination series at ≥ 16 weeks of age, in accordance with international guidelines, is critical to overcome maternally derived antibody interference. Antigenic variation appears to play a minor role in disease occurrence in this setting. CPV-2 Infection after the perceived finishing of vaccination highlights a safety gap, where the veterinary stamp occurs before the 16-week threshold required for effective protection.

**Supplementary Information:**

The online version contains supplementary material available at 10.1186/s12917-026-05403-0.

## Background

Canine parvovirus type 2 (CPV-2) is a highly contagious pathogen that causes acute, often fatal enteritis in young puppies worldwide [1]. CPV-2 is a small, non-enveloped, environmentally resistant virus belonging to the species *Protoparvovirus carnivoran1*, genus *Protoparvovirus*, family *Parvoviridae* [2]. The original CPV-2 type emerged in 1978, and since then, three antigenic variants, designated as CPV-2a, CPV-2b, and CPV-2c, have evolved, replacing the original type as the predominant virulent field strains [3]. Nevertheless, the original type remains included in most commercial vaccines [4].

While CPV-2 infection is routinely diagnosed using antigen detection point-of-care kits or PCR, these methods cannot reliably distinguish antigenic variants or discriminate between field and vaccinal strains, which is crucial for both epidemiological surveillance and the clinical management of gastroenteritis cases that occur shortly after vaccination [5–7]. Consequently, sequencing of the VP2 gene and analysis of key amino acid residues are necessary for accurate strain identification [6].

Vaccination using modified live virus (MLV) remains the primary strategy for preventing CPV-2 infection [4]. According to international vaccination guidelines, including those issued by the World Small Animal Veterinary Association (WSAVA) and the American Animal Hospital Association (AAHA), CPV-2 is classified as a core vaccine, recommended for all dogs, regardless of lifestyle or geographic location, due to the severe nature of the disease [8–9]. These guidelines recommend a primary vaccination series beginning at 6–8 weeks of age, repeated every 2–4 weeks until at least 16 weeks, with particular emphasis on administering the final dose at or after 16 weeks, regardless of the total number of doses or the specific vaccinal strain used. Following finishing the primary vaccination series, a booster is recommended at 6–12 months, with subsequent revaccination every 3 years [8–9].

Despite widespread vaccination, CPV-2 infection continues to be reported in puppies worldwide, a phenomenon often attributed to the high environmental stability of the virus and the complexity of achieving protective immunity [10]. Cases of CPV-2 infection in vaccinated dogs have been documented in various geographic regions [11–19], with potential contributing factors including interference by maternally derived antibodies (MDA), suboptimal vaccination practices, exposure to virulent field strains shortly before or after vaccination, and antigenic variation among circulating CPV-2 strains [20].

Similarly, in Egypt, CPV-2 infection has been documented in vaccinated puppies [21–24]; however, the factors contributing to these cases have not been systematically studied. In the present study, we investigated determinants of CPV-2 infection in puppies whose primary vaccination series is practically considered ‘finished’ (stamped) by attending veterinarians, regardless of whether international guidelines were followed. Since laboratory-based serological confirmation of immunity after the last vaccine dose is not a routine practice in the field [8], a stamped record of finishing, when vaccination practices deviate from international recommendations, could be perceived by owners as evidence of protection, potentially influencing their behavior and increasing the risk of CPV-2 exposure. Such puppies are therefore particularly relevant for assessing the impact of routine field vaccination practices. Additionally, because CPV-2 is a rapidly evolving virus [2], viral diversity could contribute to the risk of CPV-2 infection.

Accordingly, this study aimed to investigate the role of selected aspects of routine vaccination (number of doses, vaccinal strain, and age at finishing) and viral antigenic variation in CPV-2 infection in this specific canine population.

## Materials and methods

### Ethical approval

The Institutional Animal Care and Use Committee (IACUC) at the Faculty of Veterinary Medicine, Cairo University, approved the current study (Vet CU 01102020235). Verbal informed consent was obtained from the dog owners before sampling.

### Source population, eligibility criteria, and sampling

A total of 180 puppies, representing all puppies presenting with clinical signs suggestive of canine parvoviral enteritis, including anorexia, depression, diarrhea, and vomiting (Supplementary Fig. 1), at three veterinary clinics in Giza Governorate, Egypt, during June-October 2020, constituted the source population for this study and were assessed for possible enrollment.

The eligibility and exclusion criteria for enrollment were based on strictly documented vaccination histories. Vaccination status was determined using available medical records, vaccination cards, and history-taking from owners. Based on this information, puppies were classified as having unknown or undocumented vaccination history (*n* = 47), vaccinated (*n* = 116), or non-vaccinated (*n* = 17).

Puppies with unknown or undocumented CPV-2 vaccination status were excluded to ensure reliable classification of vaccination exposure. All non-vaccinated puppies were included as a reference group to enable comparison of circulating CPV-2 strains. Among vaccinated puppies, those whose primary vaccination series had been stamped as ‘finished’ by their attending veterinarians were included (*n* = 41), whereas those still undergoing their primary vaccination series (i.e., between scheduled doses) were excluded (*n* = 75), to focus on puppies considered fully vaccinated in routine field practice. For this study, the term ‘finished’ refers solely to documented finishing of the primary vaccination series and does not necessarily correspond to international guideline definitions nor imply immunological confirmation.

Accordingly, the study population consisted of 58 puppies, including 17 non-vaccinated puppies and 41 puppies who had received a final documented veterinary stamp for their primary vaccination series (hereafter referred to as vaccinated puppies). Supplementary Fig. 2 provides a flowchart, illustrating the source population, classification of puppies according to vaccination status, and the final study population.

For vaccinated puppies, detailed information regarding vaccination practices, including the number of doses administered, vaccinal strain, and age at finishing the primary vaccination series, was extracted from vaccination cards and categorized and defined as shown in Table 1 for further analysis. Additionally, the time elapsed between the last dose and the occurrence of disease was recorded (hereafter referred to as time since last vaccination).

**Table 1:** Vaccination practice-related variables extracted from vaccination cards and their categorization

Variable	Categories	Definition
Age at finishing the primary vaccination seriesᵃ	Early finish-age	Last dose given at age < 16 weeks
	Recommended finish-age	Last dose given at age ≥ 16 weeks
Number of vaccine doses	Three doses	NA
	Four doses	NA
Vaccinal strain	Strain 154	CPV-2 original type–based, high-titer vaccine (≥ 10⁷ TCID₅₀)
	Strain NL-35	CPV-2 original type–based, low-passage, high-titer vaccine (≥ 10⁷ TCID₅₀)

ᵃAge at finishing the series was categorized according to international guidelines AAHA/WSAVA [8–9]

*NA* not applicable

Rectal swab samples were collected from all enrolled puppies and transported to the Laboratory of Internal Medicine and Infectious Diseases, Faculty of Veterinary Medicine, Cairo University (FVM/CU), where they were stored at − 20 °C until further processing.

### Molecular detection of CPV-2

Total DNA was extracted from rectal swabs using the QIAamp Fast DNA Stool Mini Kit (Qiagen, Germany), following the manufacturer’s instructions with the “Protocol: Isolation of DNA from Stool for Pathogen Detection,” optimized for pathogen DNA extraction.

Detection of CPV-2 DNA was performed using conventional PCR targeting a partial region of the VP2 gene; the primers used were previously reported by Decaro et al., 2008 [25], and the details of these primers are clarified in Table 2. Each 25 µL PCR reaction contained: 12.5 µL of 2× PCR Master Mix (Thermo Fisher Scientific, USA), 1 µL of forward primer (10 µM), 1 µL of reverse primer (10 µM), 5 µL of extracted DNA, and 5.5 µL of nuclease-free water to reach the final volume. Each PCR run included PCR-grade water as a negative control and CPV-2c (strain VetCU-14/2019, GenBank accession number MT636872 [21]) as a positive control. Thermal cycling was performed in a Biometra thermal cycler with the following conditions: initial denaturation at 94 °C for 5 min, followed by 35 cycles of denaturation at 94 °C for 30 s, annealing at 55 °C for 30 s, and extension at 72 °C for 1 min, and with a final extension at 72 °C for 7 min [25].

**Table 2:** Primers used for conventional PCR detection of CPV-2

Name	Sequence 5’-3’ (polarity)	positionᵃ	Amplicon size	Reference
CPV3381-F	CCATGGAAACCAACCATACC (+)	3381–3400	717 bp	[20]
CPV4116-R	AGTTAATTCCTGTTTTACCTCCAA (-)	4093–4116		

ᵃ Oligonucleotide positions are referred to the genomic sequence of CPV-2 strain CPV-b (GenBank accession no. M38245)

PCR products were separated by electrophoresis on a 1.5% agarose gel stained with ethidium bromide and visualized under UV illumination using a Gel Doc XR system (Bio-Rad, Milan, Italy).

### Partial sequencing and phylogenetic analysis of the VP2 gene

Amplicons from all PCR-positive samples were purified from agarose gels using the QIAquick^®^ Gel Extraction Kit (Qiagen, Germany), following the manufacturer’s instructions. Purified amplicons were sequenced bidirectionally using the same PCR primers with the BigDye™ Direct Cycle Sequencing Kit (ThermoFisher, USA). The obtained nucleotide sequences were checked for quality, edited, assembled, and translated to amino acid sequences using BioEdit version 7.0.9.1.

Viral identity was determined based on key amino acid residues at positions 297, 300, 305, 323, 326, and 426 of VP2. Residue 323 was used to differentiate between CPV-2 (Asn) and feline parvovirus (FPV) (Asp). The original CPV-2 type (vaccinal strain) was distinguished from field CPV-2 variants by residues 300 and 305 as follows: 300 (Ala in the original type, Gly in variants) and 305 (Asp in the original type, Tyr in variants). Residue 426 was used to classify CPV-2a (Asn), CPV-2b (Asp), and CPV-2c (Glu), while residue 297 was used to distinguish “new” CPV-2a and CPV-2b from older strains (Ser→Ala) [26].

The nucleotide and deduced amino acid sequences obtained in this study were grouped into nucleotide sequence types (nSTs) and amino acid sequence types (aaSTs), with each nST or aaST representing a set of identical sequences.

Reference sequences for CPV-2 and FPV were retrieved from GenBank to guide viral typing and to examine nucleotide and amino acid variations of sequences obtained in this study. These included the original CPV-2 type (M38245.1), CPV-2a old (M24003.1) and new (AY742953), CPV-2b old (M74849.1) and new (AY742955), CPV-2c (FJ222821.1), CPV-2 vaccinal strains 154 (ON479058) and NL-35 (ON479057), and FPV (M38246).

**Fig. 1:**
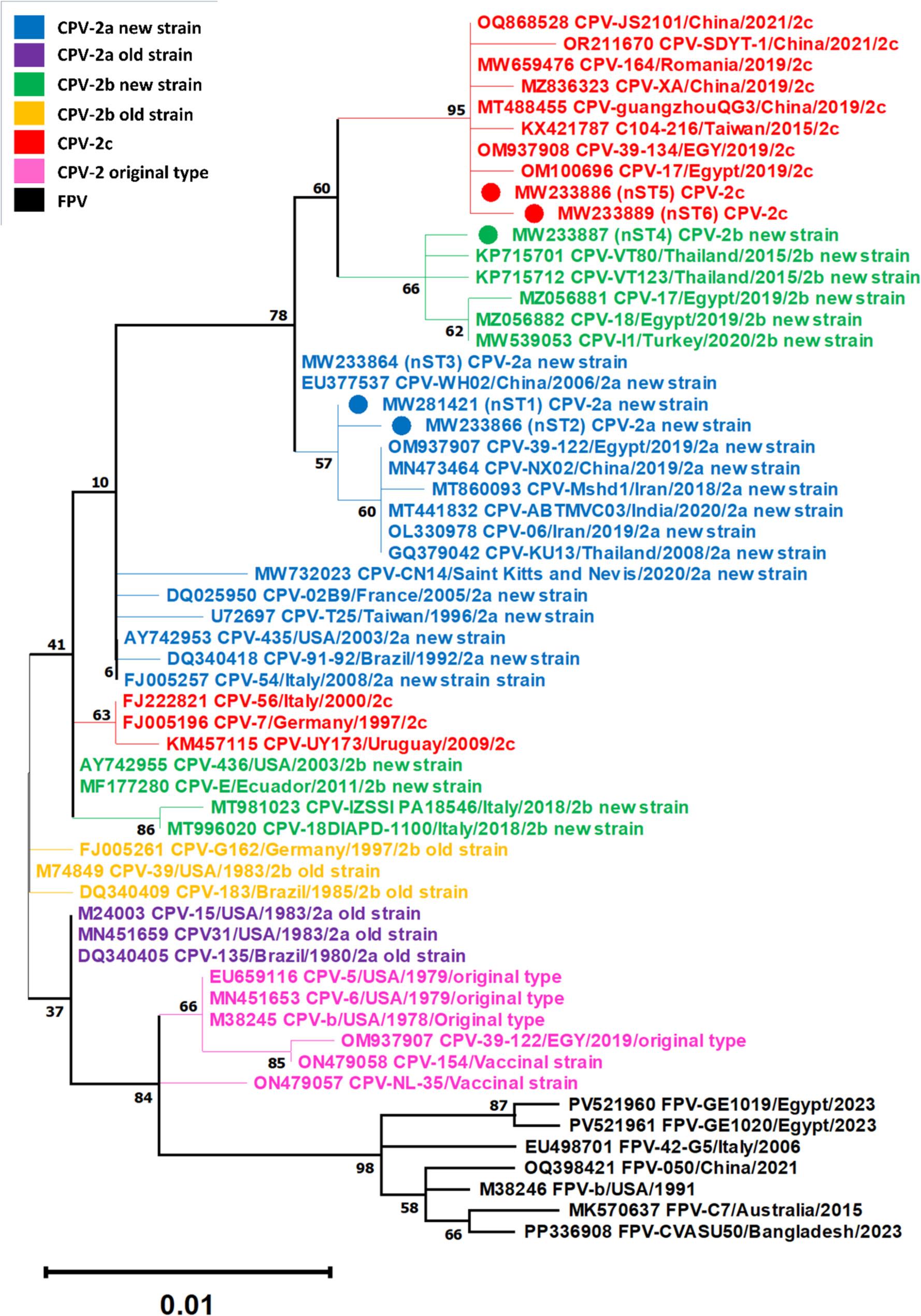
Maximum likelihood phylogenetic tree based on partial VP2 gene sequences (681 bp) of CPV-2 obtained in this study, together with reference CPV-2 and FPV sequences retrieved from GenBank. Each of the six Egyptian nucleotide sequence types (nSTs) is represented by a single sequence; the GenBank accession numbers of all sequences included within each nST are provided in the legend of Table 5. The scale bar indicates the number of nucleotide substitutions per site. Phylogenetic analysis showed that all sequences generated in this study clustered within clades corresponding to circulating CPV-2 field variants (CPV-2a new strain: nST1–3, blue circles; CPV-2b new strain: nST4, green circle; CPV-2c: nST5–6, red circles) and were clearly separated from vaccinal strains.

To further confirm viral identity, phylogenetic analysis of the partial VP2 gene sequences obtained in this study was performed together with 52 reference sequences, selected from GenBank, using the maximum likelihood (ML) method implemented in MEGA version 11. The Tamura 3-parameter model with a discrete gamma distribution (T92 + G) was selected as the best-fitting nucleotide substitution model using the “Find Best DNA Model” tool in MEGA. Branch support was evaluated using the bootstrap method with 1000 replicates. The resulting phylogenetic tree was visualized and edited using FigTree version 1.4.4. GenBank accession numbers, viral identity, country of origin, and year of collection for reference sequences are indicated on the tree (see Results; Fig. 1).

### Statistical analysis

All statistical analyses were performed using Statistical Package for the Social Sciences (SPSS) software. Associations between different aspects of vaccination practice (variables listed in Table 1) and CPV-2 PCR positivity among vaccinated puppies were assessed using Fisher’s exact test when expected cell counts were < 5 and chi-square test when expected cell counts were ≥ 5. Variables with P values < 0.20 in univariate analysis were predefined as eligible candidates for multivariable logistic regression, in which a P value < 0.05 was considered statistically significant for all hypothesis tests. Odds ratios (ORs) with 95% confidence intervals (CIs) were calculated for statistically significant variables.

To explore whether the timing of disease onset relative to the last vaccination differed between puppies finishing the primary vaccination series early versus at the recommended age, a cross-tabulation of time since last vaccination (in weeks) against age-at-finish groups was performed. Fisher’s exact test was applied to assess statistical significance, with *P* < 0.05 considered significant.

To assess the impact of antigenic variation on CPV-2 infection in vaccinated puppies, the distribution of CPV-2 antigenic variants was compared between vaccinated and non-vaccinated CPV-2–positive puppies using Fisher’s exact test, as expected cell counts were < 5. A P value < 0.05 was considered statistically significant. Finally, age distributions were compared between vaccinated and unvaccinated puppies to exclude age as a potential confounding factor in the inter-group comparison, using the Mann–Whitney U test.

## Results

### Molecular detection of CPV-2

Among the 58 puppies that met the eligibility criteria, conventional PCR detected CPV-2 infection in 41 (70.8%), producing amplicons of the expected size (Supplementary Fig. 2). This positive cohort included 28 of 41 (68.3%) vaccinated puppies and 13 of 17 (76.5%) non-vaccinated puppies.

### Associations between vaccination practices and CPV-2 PCR positivity

Data regarding the vaccination history of each vaccinated puppy, including the number of administered doses, vaccinal strain, age at finishing the primary vaccination series, vaccination interval, and time since last vaccination, are provided in Supplementary Table 1.

Among vaccinated puppies, 31 had finished the primary vaccination series before 16 weeks of age, whereas 10 had finished the series at ≥ 16 weeks. All PCR-positive cases occurred in the early-finish group (28/31, 90.3%), while no CPV-2 infection was detected among puppies finishing the series at the recommended age (0/10, 0%). Early finishing of the primary vaccination series was significantly associated with CPV-2 PCR positivity (*P* < 0.001).

Vaccinated puppies had received either three (*n* = 27) or four (*n* = 14) doses of commercial MLV vaccines, with no statistically significant difference in PCR positivity between the two groups (*P* = 0.26). Puppies were vaccinated with either a strain 154-based vaccine (*n* = 8) or an NL-35-based vaccine (*n* = 33); the vaccinal strain used was not significantly associated with CPV-2 PCR positivity (*P* = 0.69).

Only age at finishing the primary vaccination series met the predefined screening criterion for multivariable analysis. Given the limited sample size, the absence of additional eligible covariates, and complete separation of outcomes across age categories, multivariable logistic regression was not performed. Results are therefore presented from univariate analyses only. Associations between vaccination practice–related variables and CPV-2 PCR positivity are summarized in Table 3.

**Table 3:** Associations between vaccination practice-related variables and CPV-2 PCR positivity

Variable	Categories	*n*	CPV-2 PCR Positivity *n* (%)	*P* value	Odds ratio (95% CI)
Age at finishing the primary series	Early finish age (< 16 weeks)	31	28 (90.3)	< 0.001	171 (8-3598)
	Recommended finish age (≥ 16 weeks)	10	0 (0)		
Number of vaccine doses	Three doses	27	20 (75)	0.26	-
	Four doses	14	8 (53.8)		
Vaccinal strain	Strain 154	33	23 (65.5)	0.69	-
	Strain NL-35	8	5 (75)		

All vaccinated puppies received their final vaccine dose within one month prior to the onset of clinical signs. Specifically, disease onset occurred 1 week after vaccination in 6 puppies, 2 weeks in 11 puppies, 3 weeks in 15 puppies, and 4 weeks in 9 puppies. Cross-tabulation of time since last vaccination by age-at-finish group showed no statistically significant difference (P = 0.30; Table 4).

**Table 4:** Cross-tabulation of time since last vaccination by age-at-finishing group

		Time since last vaccination			
		1 weeks	2 weeks	3 weeks	4 weeks
Age-at-finish group	Early finish (< 16 weeks)	6	7	12	6
	Recommended finish (≥ 16 weeks)	0	4	3	3

### CPV-2 antigenic variant distribution in vaccinated and non-vaccinated puppies

Partial VP2 gene sequences were obtained from all 41 PCR-positive samples (28 vaccinated, 13 non-vaccinated) and deposited in GenBank with the following accession numbers: MW233859.2–MW233892.2 and MW281416.2–MW281422.2.

Amino acid sequence analysis at VP2 residues 297, 300, 305, and 426 identified 35 samples as new CPV-2a (Asn), three as new CPV-2b (Asp), and three as CPV-2c (Glu) variants. These classifications were further supported by phylogenetic analysis (Fig. 1). Accordingly, detected viral strains in all PCR-positive vaccinated puppies were identified as circulating field strains rather than the administered vaccinal strains.

Nucleotide sequences obtained in this study showed 13 nucleotide substitutions compared with the reference original CPV-2 type (CPV-b; M38245), including three synonymous and ten non-synonymous mutations. These variations classified the sequences into six distinct nSTs: CPV-2a was represented by three nSTs (nST1–nST3), CPV-2b by one nST (nST4), and CPV-2c by two nSTs (nST5–nST6). Table 5 presents the specific VP2 nucleotide positions of these substitutions, the identity of the changes, and the GenBank accession numbers of sequences in each nST.

**Table 5:**
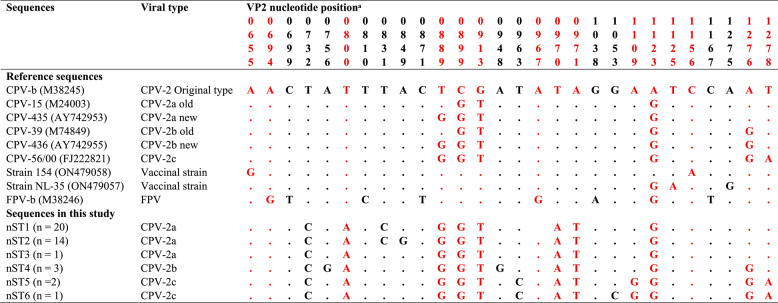
Nucleotide sequence types (nSTs) of CPV-2 identified in this study and site-specific VP2 nucleotide differences compared with reference strains

^a^ Nucleotide positions correspond to the VP2 gene and are numbered according to the CPV-b original type reference sequence

Dots (.) indicate nucleotide identity with CPV-2 original type reference strain (CPV-b; M38245)

Non-synonymous nucleotide substitutions are highlighted in red, resulting in 13 amino acid changes across the alignment (see Table 6)

nST1: MW233861–MW233863, MW233865, MW233867, MW233868, MW233871, MW233875–MW233883, MW233890–MW233892, MW281421; nST2: MW233866, MW233869–MW233870, MW233872–MW233874, MW233884–MW233885, MW281416–MW281420, MW281422; nST3: MW233864; nST4: MW233859–MW233860, MW233887; nST5: MW233886, MW233888; nST6: MW233889

The ten non-synonymous mutations resulted in eight amino acid substitutions, which classified the deduced amino acid sequences into three aaSTs: aaST1 comprising all CPV-2a new strain sequences, aaST2 comprising all CPV-2b new strain sequences, and aaST3 comprising all CPV-2c sequences (Table 6). Supplementary Figs. 4 and 5 show multiple sequence alignments of the Egyptian nSTs and aaSTs together with reference sequences, covering the full analyzed region of the VP2 gene (681 bp) and the deduced VP2 protein sequences (217 amino acids), respectively.

**Table 6:** Amino acid sequence types (aaSTs) of CPV-2 identified in this study and site-specific VP2 amino acid residues compared with reference strains

Sequences	Viral type	VP2 amino acid residues											
		219	232	267	297	300	305	323	324	370	375	386	426
Reference sequences													
CPV-b (M38245)	CPV-2 Original type	Ile	Ile	Phe	Ser	Ala	Asp	Asn	Tyr	Gln	Asn	Gln	Asn
CPV-15 (M24003)	CPV-2a old	.	.	.	.	Gly	Tyr	.	.	.	Asp	.	.
CPV-435 (AY742953)	CPV-2a new	.	.	.	Ala	Gly	Tyr	.	.	.	Asp	.	.
CPV-39 (M74849)	CPV-2b old	.	.	.	.	Gly	Tyr	.	.	.	Asp	.	Asp
CPV-436 (AY742955)	CPV-2b new	.	.	.	Ala	Gly	Tyr	.	.	.	Asp	.	Asp
CPV-56/00 (FJ222821)	CPV-2c	.	.	.	Ala	Gly	Tyr	.	.	.	Asp	.	Glu
Strain 154 (ON479058)	Vaccinal strain	Val	.	.	.	Gly	Tyr	.	.	.	.	Lys	.
Strain NL-35 (ON479057)	Vaccinal strain	.	.	.	.	Gly	Tyr	.	.	.	Glu	.	.
FPV-b (M38246)	FPV	.	Val	.	.	Gly	Tyr	Asp	.	.	Asp	.	.
Sequences in this study													
aaST1 (*n* = 32)	CPV-2a	.	.	Tyr	Ala	Gly	Tyr		Ile	.	Asp	.	.
aaST3 (*n* = 3)	CPV-2b	.	.	Tyr	Ala	Gly	Tyr	.	Ile	.	Asp	.	Asp
aaST4 (*n* = 3)	CPV-2c	.	.	Tyr	Ala	Gly	Tyr	.	Ile	Arg	Asp	.	Glu

Dots (.) indicate amino acid (aa) identity with the CPV-2 original type reference strain (CPV-b; M38245). Amino acid positions correspond to the VP2 protein and are numbered according to the CPV-2 original type reference strain (CPV-b; M38245). Amino acid residues at positions 297, 300, 305, 323, and 426 are key residues for viral typing, as described in the Materials and Methods

Table 7 presents the distribution of CPV-2 antigenic variants among vaccinated and non-vaccinated puppies; no statistically significant difference was observed between the two groups with respect to this distribution. The median age at admission was 16 weeks for both vaccinated and non-vaccinated puppies, with no significant difference between groups (P = 0.12), supporting the comparability of these groups.

**Table 7:** Distribution of CPV-2 antigenic variants among vaccinated and non-vaccinated puppies

		CPV-2 antigenic variants			*n*
		CPV-2a	CPV-2b	CPV-2c	
Vaccination status	Vaccinatedᵃ	24	1	3	28
	Non-vaccinated	11	2	0	13
*N*		35	3	3	41

There was no statistically significant difference in the distribution of CPV-2 antigenic variants between vaccinated and non-vaccinated puppies (*P* = 0.16038)

## Discussion

This study investigated factors associated with CPV-2 infection in puppies whose primary vaccination series was considered finished under routine field practice. Because clinical disease occurred in close temporal association with vaccination in all puppies, an essential first step was to determine whether PCR positivity represented true infection with circulating CPV-2 field strains or merely the detection of vaccine-derived virus. This distinction is critical, as viremia, fecal shedding, and tissue localization of CPV-2 vaccinal strains following MLV vaccination have been demonstrated using molecular techniques [7, 27–29]. All examined viral strains were typed, through sequence analysis, as CPV-2 antigenic variants, indicating that the PCR detected circulating field strains rather than the administered vaccine virus.

Having confirmed that disease in vaccinated puppies was caused by field strains, vaccination practices were examined to identify factors associated with CPV-2 infection. Early finishing of the primary vaccination series (before 16 weeks of age) was significantly associated with CPV-2 infection in vaccinated puppies. Consistent with this finding, a previous retrospective study from Australia reported that the age at last vaccination was associated with immunization failure in puppies [30]. By approximately 16 weeks of age, MDA titers are generally low enough to allow an effective vaccine response, which is why international guidelines recommend finishing the primary vaccination series no earlier than this age, ensuring that at least one dose induces protective immunity even if earlier doses were neutralized by MDA [8–9]. Collectively, these findings suggest that CPV-2 infection despite vaccination in the present study likely reflects a probable MDA-mediated vaccine neutralization due to a suboptimal timing of primary vaccination series finishing.

While this explanation is plausible because MDA interference is recognized as the most common cause of immunization failure in puppies [8–9, 20], MDA titers were not measured in this study, and the hypothesis, therefore, remains speculative. Furthermore, other factors, such as individual variation in MDA half-life, breed-related differences in immune response, environmental exposures, and housing conditions, could also contribute to the risk of CPV-2 infection in puppies with early finishing of the primary vaccination series.

Given the emphasis of international guidelines on finishing the primary vaccination series at the recommended age, understanding the extent of guideline compliance is critical. However, data on adherence to these guidelines among veterinary practices in Egypt are lacking. Globally, systematic studies are also limited, with only one report indicating that 48.7% of veterinarians in Australia did not follow international vaccination guidelines [31]. As vaccination protocols and professional awareness continue to evolve, further studies at both local and international levels are needed to better characterize adherence patterns and their impact on immunization outcomes.

In contrast to the age at finishing the primary vaccination series, the number of vaccine doses was not associated with CPV-2 infection in vaccinated puppies in this study. For commercial MLV vaccines, multiple doses are intended not to boost an existing response but to ensure that at least one dose is effective if earlier doses were neutralized by MDA [8–9]. Therefore, the total number of doses in the primary vaccination series is not fixed and should be determined by the age at vaccination onset and the revaccination intervals used, with the sole objective of ensuring that the final dose is administered at or beyond 16 weeks of age [8–9].

Similarly, CPV-2 infection in vaccinated puppies was not associated with vaccinal strains in this study. The occurrence of CPV-2 infection despite the use of high-titer vaccines designed to shorten the MDA interference window and adherence to manufacturer-recommended protocols suggests that such vaccines may not consistently overcome MDA interference in all puppies. Supporting this, a recent experimental study reported that a high-titer vaccine (Vanguard© Plus) induced an immune response in only two of four puppies, with these two puppies having lower baseline antibody titers than the non-responders [32]. This demonstrates that even high-titer vaccines may fail to protect puppies with high MDA and cannot be expected to provide uniform protection. Moreover, the lack of a significant difference between vaccinal strains may indicate that neither product performs better in this context, with both potentially limited by MDA.

Given the limitations of high-titer low-passage vaccines in overcoming MDA in some animals, alternative vaccination strategies have been explored using novel technologies. Recombinant CPV-2/CPV-2c vaccines, incorporating a chimeric genome of CPV-2 and CPV-2c, have demonstrated protective immunity in experimental studies even in the presence of MDA [32–33]. While these vaccines offer promise for addressing one of the main causes of immunization failure, their practical implementation may be limited by factors such as cost, stability requirements, and vaccination regimen. Moreover, they have not yet been evaluated in field settings and are not currently available in Egypt. Consequently, their field impact on preventing early CPV-2 infection in puppies in this context remains to be determined.

Notably, six puppies developed clinical disease within one week of vaccination. The exact timing and source of CPV-2 infection cannot be determined. However, given the short incubation period of CPV-2 (2–7 days) [6], exposure to field strains shortly before or after the time of vaccination, combined with interference from MDA, remains a plausible explanation for disease occurrence in these puppies. The occurrence of parvoviral enteritis shortly after vaccination may lead owners to mistakenly attribute the disease to the vaccine itself [5]. However, reversion to virulence of CPV-2 vaccinal strains is rarely documented [20]. Moreover, vaccine contamination is unlikely, as vaccines from international manufacturers undergo strict quality control [20]. Supporting the rarity of vaccine-induced disease, a previous study has shown that when vaccinal strain is detected in recently vaccinated dogs, it is typically present at viral loads far lower than the high titers usually observed in clinically affected dogs [5].

Beyond vaccination practices, we studied the distribution of CPV-2 antigenic variants among vaccinated and non-vaccinated puppies to gain insights into the potential role of antigenic variation in disease occurrence. To reliably identify circulating variants, we used a previously described PCR targeting conserved regions of the VP2 gene, which has been validated to amplify all known CPV-2 variants (2a, 2b, 2c) [25, 34–37]. The difference in the distribution of CPV-2 variants not statistically significant. Given the low number of cases for some variants (CPV-2b and CPV-2c, *n* = 3 each), this result should be interpreted cautiously, as small sample sizes limit the ability to detect subtle differences. Nonetheless, the observed distribution is broadly consistent with previous reports showing similar patterns of CPV-2 variants among vaccinated and unvaccinated puppies [38], suggesting that antigenic variation might not be the main driver of CPV-2 infection in vaccinated puppies, though a contributory role cannot be completely excluded.

VP2 amino acid analysis revealed substitutions in the Egyptian field strains at residues 267, 324, and 370 compared with the vaccinal strains (Table 6). While mutations at residues 267 and 324 have been highlighted in the literature as potentially relevant to reduced vaccine protection or vaccine failure [5, 26], no experimental or epidemiological evidence currently confirms this speculation. Residue 370 is adjacent to residues 379 and 384, which are involved in transferrin receptor binding. The mutation 370Arg, observed mostly in CPV-2c and rarely in CPV-2a, may influence protein conformation or receptor interactions, potentially expanding host range. Its functional role in viral biology remains unknown, but it is unlikely to directly impact vaccination failure [39].

While the efficacy of original CPV-2 type-based vaccines against circulating variants was not directly assessed in this study, previous serological in vitro studies indicate that vaccines based on the original CPV-2 type elicit cross-neutralizing antibodies against multiple field variants [40]. While serological neutralization in vitro does not necessarily guarantee clinical protection in a living host, challenge-protection experiments, the gold standard for assessing vaccine efficacy, have demonstrated that CPV-2-based vaccines confer robust protection against clinical disease when puppies are challenged with virulent CPV-2a, 2b, or 2c variants [41–44]. These findings support the notion that factors other than antigenic variation, such as MDA and timing of vaccination, are likely more important contributors to CPV-2 infection in vaccinated puppies.

This study has several limitations. First, the relatively small sample size may have limited statistical power to detect subtle associations, and the observational design of the study does not allow definitive conclusions about causality. Second, other factors, which may influence immune response to vaccination, such as non-responder puppies, host genetic factors, improper vaccine handling, or manufacturing issues, were not assessed and therefore cannot be excluded. Third, CPV-2 detection was performed using conventional qualitative PCR, and viral loads were not measured, making it impossible to compare viral loads between vaccinated and unvaccinated puppies or assess their relationship with immune protection. Fourth, molecular typing relied on selected key amino acid residues within the VP2 gene (positions 297, 300, 305, and 426). The inclusion of additional VP2 residues or full-length VP2 sequencing could further improve resolution, especially in situations involving vaccine-derived or reverted strains. However, large-scale global sequence analyses indicate that vaccine-like CPV-2 strains are uncommon in the field [5, 26]. Moreover, because only a partial region of VP2 was analyzed, the presence of recombinant viruses containing FPV–derived genomic regions outside the sequenced fragment cannot be completely excluded. However, large-scale global sequence analyses indicate that FPV is rarely detected in dogs, suggesting that opportunities for cross-species coinfection and subsequent recombination events are uncommon in the field [30].

Future studies could include larger-scale prospective investigations in Egypt that correlate MDA titers with vaccine failure, while simultaneously accounting for other causes of immunization failure, and incorporating quantitative PCR to measure viral loads and assess their relationship with immune protection and clinical outcomes. Such studies would provide a more detailed understanding of early CPV-2 infection dynamics and help optimize vaccination strategies in puppies.

## Conclusion

This study shows that the timing of primary vaccination series finishing, rather than vaccinal strain, formulation, or number of doses, is the key factor influencing immunization success in young puppies, likely by reducing MDA-mediated interference. finishing the final dose at or beyond 16 weeks of age is therefore essential. The findings of this study suggest that antigenic variation is unlikely to be the primary driver of CPV-2 infection in vaccinated puppies. Future research should focus on larger-scale studies and improved vaccination strategies to overcome maternal antibody interference and optimize early protection against CPV-2.

## Supplementary Information

Supplementary Material 1.

Supplementary Material 2.

Supplementary Material 3.

Supplementary Material 4.

Supplementary Material 5.

Supplementary Material 6.

Supplementary Material 7.

## Data Availability

All data generated and analyzed in this study were reported in the manuscript and Supplementary Table 1. Forty-one partial CPV-2 VP2 gene sequences were generated in this study and deposited in GenBank under the following accession numbers: MW233859.2–MW233892.2 and MW281416.2–MW281422.2.
