# Supplementary material for: Canine parvovirus type 2 infection in vaccinated puppies: role of vaccination practices and viral antigenic variation
Source: BMC Vet Res. 2026 Mar 26;22:214. doi: 10.1186/s12917-026-05403-0 (PMC13063580; doi:10.1186/s12917-026-05403-0)
Supplement: Supplementary file 1 — Supplementary Material 1. [file 12917_2026_5403_MOESM1_ESM.docx]

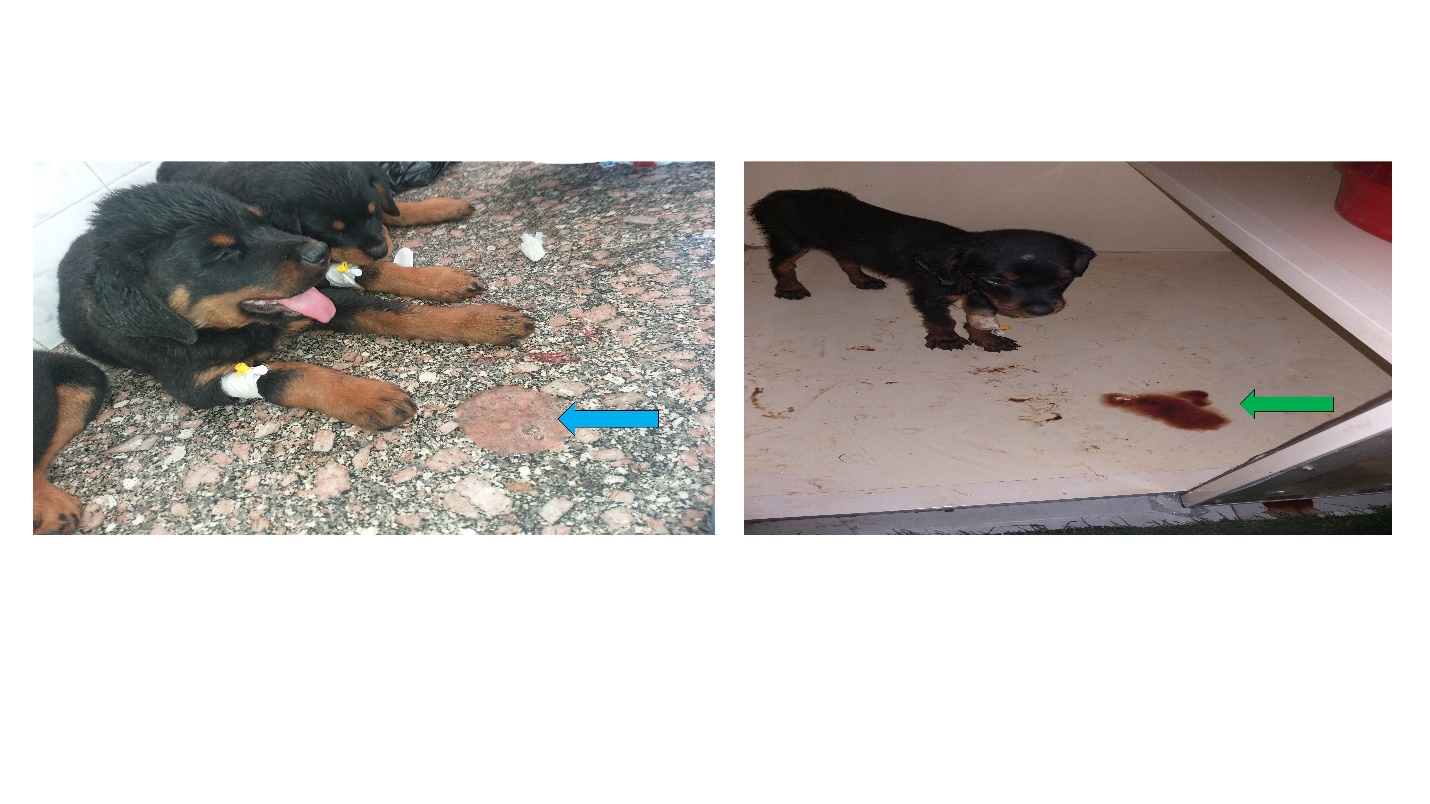
**Supplementary Figure 1.** Two puppies exhibiting clinical signs consistent with canine parvoviral enteritis. The puppy on the left shows bloody vomitus (blue arrow), whereas the puppy on the right presents with bloody diarrhea (green arrow). Both animals appear dull and severely dehydrated.
