# Supplementary material for: Canine parvovirus type 2 infection in vaccinated puppies: role of vaccination practices and viral antigenic variation
Source: BMC Vet Res. 2026 Mar 26;22:214. doi: 10.1186/s12917-026-05403-0 (PMC13063580; doi:10.1186/s12917-026-05403-0)
Supplement: Supplementary file 3 — Supplementary Material 3. [file 12917_2026_5403_MOESM3_ESM.docx]

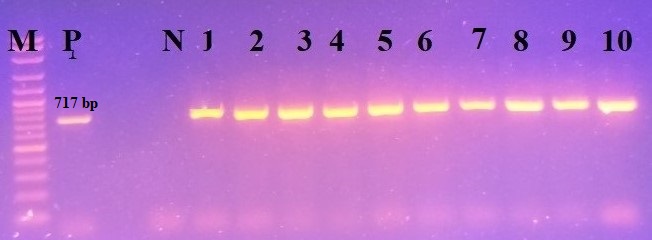


**Supplementary Figure 3.** Conventional PCR using primer pair CPV3381-F / CPV4116-R. A positive reaction is indicated by an amplicon of 717 bp. Lane M: 100 bp DNA ladder; P: positive control (CPV-2c strain VetCU-14/2019, GenBank accession number MT636872); N: negative control (PCR-grade water); lanes 1–10: positive samples from this study.
