# Supplementary material for: Canine parvovirus type 2 infection in vaccinated puppies: role of vaccination practices and viral antigenic variation
Source: BMC Vet Res. 2026 Mar 26;22:214. doi: 10.1186/s12917-026-05403-0 (PMC13063580; doi:10.1186/s12917-026-05403-0)
Supplement: Supplementary file 4 — Supplementary Material 4. [file 12917_2026_5403_MOESM4_ESM.docx]

**Supplementary Figure 4.** Multiple nucleotide sequence alignment of Partial VP2 gene sequences (681 bp) of canine parvovirus type 2 (CPV-2) obtained in this study with reference strains.

Asterisks (*) indicate reference sequences, including CPV-b (original type; accession number M38245), CPV-15 (CPV-2a old strain; M24003), CPV-435 (CPV-2a new strain; AY742953), CPV-39 (CPV-2b old strain; M74849), CPV-436 (CPV-2b new strain; AY742955), CPV-56/00 (CPV-2c; FJ222821), strain 154 (vaccinal strain; ON479058), strain NL-35 (vaccinal strain; ON479057), and FPV-b (feline parvovirus; M38246).

Egyptian sequences were grouped into identical nucleotide sequence types (nSTs) as follows: **nST1**, comprising 20 CPV-2a new strain sequences (MW233861–MW233863, MW233865, MW233867–MW233868, MW233871, MW233875–MW233883, MW233890–MW233892, MW281421); **nST2**, comprising 14 CPV-2a new strain sequences (MW233866, MW233869–MW233870, MW233872–MW233874, MW233884–MW233885, MW281416–MW281420, MW281422); **nST3**, comprising one CPV-2a new strain sequence (MW233864); **nST4**, comprising three CPV-2b new strain sequences (MW233859–MW233860, MW233887); **nST5**, comprising two CPV-2c sequences (MW233886, MW233888); and **nST6**, comprising one CPV-2c sequence (MW233889).

Dots (.) indicate amino acid (aa) identity with the original CPV-2 type reference strain (CPV-b).

Nucleotide positions shown on the alignment ruler are numbered according to the CPV-b reference strain.
