# Supplementary material for: Canine parvovirus type 2 infection in vaccinated puppies: role of vaccination practices and viral antigenic variation
Source: BMC Vet Res. 2026 Mar 26;22:214. doi: 10.1186/s12917-026-05403-0 (PMC13063580; doi:10.1186/s12917-026-05403-0)
Supplement: Supplementary file 5 — Supplementary Material 5. [file 12917_2026_5403_MOESM5_ESM.docx]

**Supplementary Figure 5.** Multiple amino acid sequence alignment of canine parvovirus type 2 (CPV-2) VP2 sequences (227 amino acids) obtained in this study together with reference strains. Asterisks (*) indicate reference sequences, including CPV-b (original type; accession number M38245), CPV-15 (CPV-2a old strain; M24003), CPV-435 (CPV-2a new strain; AY742953), CPV-39 (CPV-2b old strain; M74849), CPV-436 (CPV-2b new strain; AY742955), CPV-56/00 (CPV-2c; FJ222821), strain 154 (vaccinal strain; ON479058), strain NL-35 (vaccinal strain; ON479057), and FPV-b (feline parvovirus; M38246).
Egyptian sequences were grouped into identical amino acid sequence types (aaSTs) as follows: **aaST1 contains all 35 CPV-2a new strain sequences(**MW233861–MW233885, MW233890–MW233892, MW281416–MW281422); **aaST2 contains all three CPV-2b new strain sequences** (MW233859–MW233860, MW233887); **aaST3** **contains all three CPV-2c strain sequences** MW233886, MW233888–MW233889.

Dots (.) indicate amino acid (aa) identity with the original CPV-2 type reference strain (CPV-b).

Amino acid positions shown on the alignment ruler are numbered according to the CPV-b reference strain.
