# Supplementary material for: Canine parvovirus type 2 infection in vaccinated puppies: role of vaccination practices and viral antigenic variation
Source: BMC Vet Res. 2026 Mar 26;22:214. doi: 10.1186/s12917-026-05403-0 (PMC13063580; doi:10.1186/s12917-026-05403-0)
Supplement: Supplementary file 6 — Supplementary Material 6. [file 12917_2026_5403_MOESM6_ESM.docx]

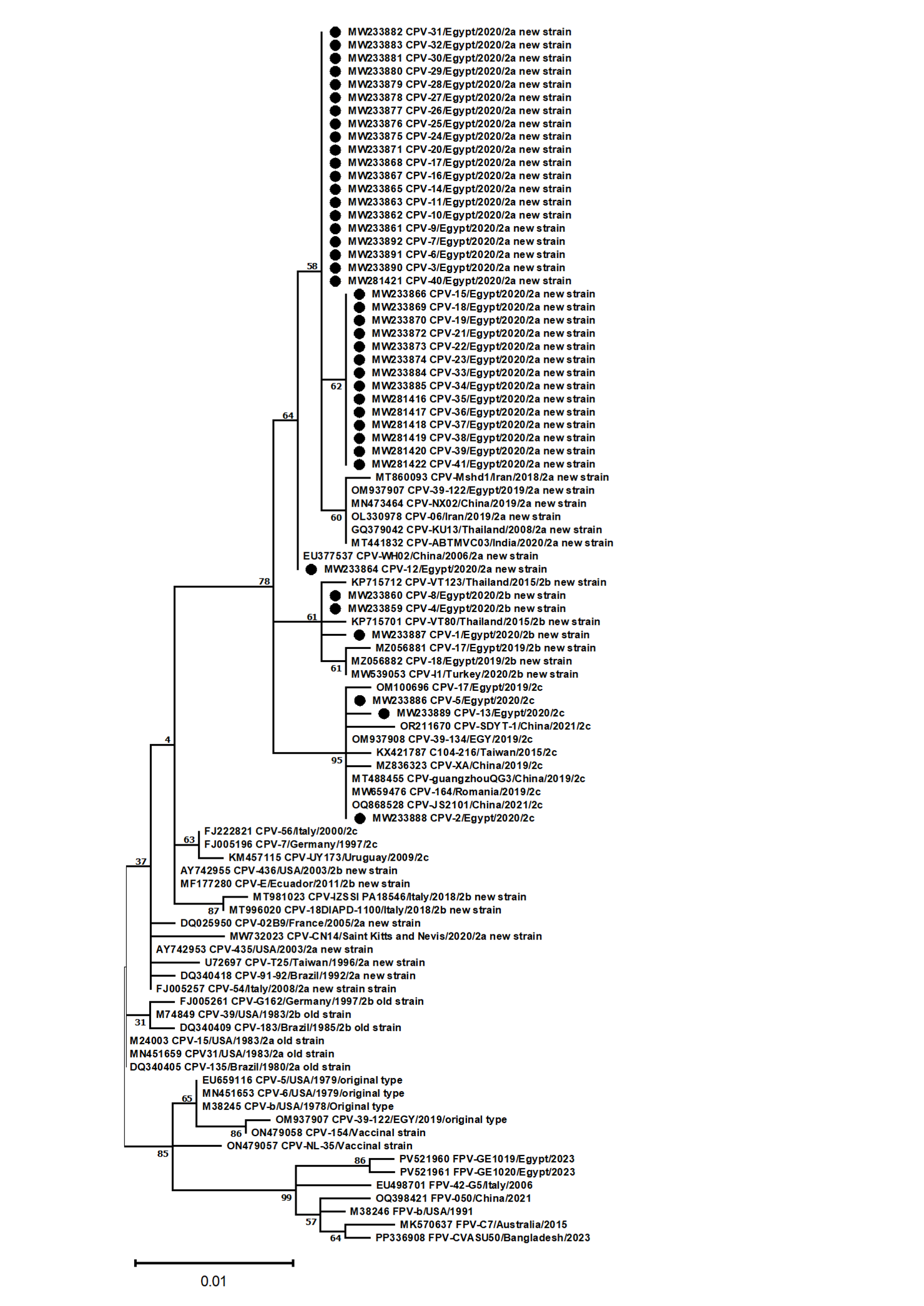


**Supplementary Figure 6.** Maximum likelihood phylogenetic tree based on partial VP2 gene sequences (681 bp) of CPV-2 obtained in this study (labelled with black circles), together with reference CPV-2 and FPV sequences retrieved from GenBank. The scale bar indicates the number of nucleotide substitutions per site. Phylogenetic analysis showed that all sequences generated in this study clustered within clades corresponding to circulating CPV-2 field variants (CPV-2a new strain, CPV-2b new strain, and CPV-2c) and were clearly separated from vaccinal strains.
